# Plasma biomarkers in patients with familial cavernous malformation and their first-degree relatives

**DOI:** 10.21203/rs.3.rs-4545797/v1

**Published:** 2024-07-01

**Authors:** Chunwang Li, Shuna Huang, Qixuan Li, Lingyun Zhuo, Yaqing Kang, Penghui Liu, Weilin Huang, Ke Ma, Xinru Lin, Weiheng Zhuang, Darong Chen, Huimin Wang, Lingjun Yan, Dengliang Wang, Yuanxiang Lin, Dezhi Kang, Fuxin Lin

**Affiliations:** First Affiliated Hospital of Fujian Medical University; First Affiliated Hospital of Fujian Medical University; First Affiliated Hospital of Fujian Medical University; First Affiliated Hospital of Fujian Medical University; First Affiliated Hospital of Fujian Medical University; First Affiliated Hospital of Fujian Medical University; First Affiliated Hospital of Fujian Medical University; First Affiliated Hospital of Fujian Medical University; First Affiliated Hospital of Fujian Medical University; First Affiliated Hospital of Fujian Medical University; First Affiliated Hospital of Fujian Medical University; First Affiliated Hospital of Fujian Medical University; First Affiliated Hospital of Fujian Medical University; First Affiliated Hospital of Fujian Medical University; First Affiliated Hospital of Fujian Medical University; First Affiliated Hospital of Fujian Medical University; First Affiliated Hospital of Fujian Medical University

**Keywords:** cerebral cavernous malformations, familial cerebral cavernous malformations, plasma biomarkers, vascular malformations, chronic disease aggressiveness, autosomal dominant inheritance

## Abstract

**Background:**

We aimed to explore the differences in plasma biomarker levels between patients with familial cerebral cavernous malformations (FCCM) and their healthy first-degree relatives (FDRs) and between FCCM patients with and without severe chronic disease aggressiveness (CDA).

**Methods:**

Magnetic resonance imaging (MRI) scanning and genetic testing was performed in patients with multiple CCMs and their FDRs. Sixty-seven plasma biomarkers were tested using a customised multiplex bead immunoassay kit. Univariate and multivariate unconditional logistic regression analyses were conducted to determine the associations between plasma factors and the risk of developing FCCM and severe CDA. Receiver operating characteristic (ROC) curves were generated for each independent risk factor.

**Results:**

Plasma factors of 37 patients with FCCM and 37 FDRs were examined. Low CD31 (*P* < 0.001) and BDNF levels (*P* = 0.013) were independent risk factors for FCCM. The best model was achieved by combining the results of CD31 and BDNF (AUC = 0.845, sensitivity 0.838, specificity 0.784, cutoff score − 4.295) to distinguish patients with FCCM from healthy FDRs. Low serpin E1/PAI-1 (*P* = 0.011) and high ROBO4 levels (*P* = 0.013) were independent risk factors for severe CDA in patients with FCCM. The best model was achieved by combining the results of E1/PAI-1 and ROBO4 levels (AUC = 0.913, sensitivity 1.000, specificity 0.760, cutoff score – 0.525) to identify patients with FCCM and severe CDA.

**Conclusions:**

The plasma concentrations of CD31 and BDNF seem to be lower in patients with FCCM than in their healthy FDRs. Low serpin E1/PAI-1 and high ROBO4 concentrations may be correlated with high lesion burden and risk of recurrent bleeding.

## Introduction

Cerebral cavernous malformations (CCMs) are abnormally clustered, dilated capillary caverns lined by a single layer of leaky endothelium^1^. Most CCM cases comprise sporadic CCMs. Hereditary or familial CCM (FCCM) is caused by an autosomal dominant inherited genetic mutation associated with multiple lesions^2^. Three protein-encoding genes, viz.*CCM1/KRIT1,CCM2*/malcavernin, and *CCM3/PDCD10*, are known to cause FCCM^3,4^. However, the clinical behavior of CCM remains unpredictable. Some patients remain asymptomatic, while others are disabled due to recurrent bleeding or a high lesion burden or a combination of both^5^. Early detection using biomarkers may improve the outcome of patients with CCM. However, the molecular mechanisms influencing and predictive biomarker for chronic or acute disease severity remain largely unknown.

Based on recent discoveries that implicate angiogenic, immune, and inflammatory mechanisms in CCM, some authors have proposed that serum biomarkers may reflect chronic or acute disease activity^6^. Girard et al. pioneered the study of the predictive value of a panel of 24 candidate plasma biomarkers, each with a reported role in the physiopathology of CCMs, to predict clinically relevant disease activity^7,8^. According to their results, the participants who had experienced symptomatic lesional hemorrhagic expansion had low sCD14, IL-6, and VEGF levels and high IL-1 and sROBO4 plasma levels^8^. Recently, Roberto Latini et al revealed sROBO4, thrombomodulin (TM) and CRP can predict incident adverse clinical events^9^. However, no significant association was observed between the burden of CCMs and biomarker plasma levels in the FCCM cohort; the predictive model of lesional activity was not derived from a pure FCCM cohort. However, familial cases with germline mutations are generally associated with a greater lesion burden and hemorrhagic risk.

Therefore, this study aimed to explore the differences in plasma biomarker levels between patients with FCCM and their healthy first-degree blood relatives (FDRs) and between FCCM patients with mild and severe chronic disease aggressiveness (CDA) to identify and stratify patients with FCCM using a new panel of 67 selected candidate plasma biomarkers.

## Methods

### Study objects

Patient with multiple CCM and their FDRs were enrolled our studies in the First Affiliated Hospital of Fujian Medical University between October 2020 and August 2021. Patients with multiple CCM were enrolled and FDRs screening procedures were previously described^10^. Patients with multiple CCMs and their FDRs were recruited after providing written informed consent for brain MRI scanning, blood sample collection and gene testing. According to the currently accepted categorization^11–13^, familial cases were defined as CCM patients with germline loss-of-function mutations in the CCM complex proteins or with a family history of CCM in FDRs. In an FCCM family, FDRs of either the proband or newly identified CCM patients were screened by MRI.

Based on the pedigree investigation, brain MRI scanning, and whole-exome sequencing (WES), the probands and FDRs with CCM were assigned to the CCM group, and healthy FDRs were assigned to the non-CCM group. The exclusion criteria were as follows: patients (1) without brain MRI or SWI sequencing; (2) with suspected CCM; (3) with arteriovenous malformation (AVM); (4) with acute intra or extra hemorrhage (≤ 1 month); or (5) without serum samples.

Our manuscript was structured in accordance with the Strengthening the Reporting of Observational Studies in Epidemiology (STROBE) reporting guidelines for cohort studies^14^.All performed in accordance with the Declaration of Helsinki and approved by the First Affiliated Hospital of Fujian Medical University Institutional Review Board (IRB; approval number: [2018] No. 003).

### Screening of participants, collection of clinical information and definition of severe chronic disease aggressiveness

**Figure 1:**
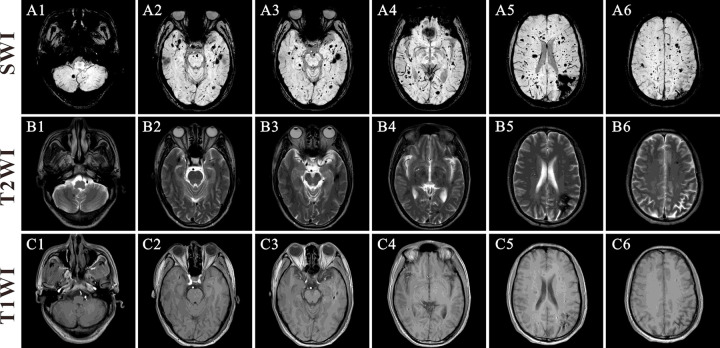
Brain magnetic resonance imaging of a typical patient with high burden of FCCMs. A1–6 were SWI. B1–6 were T2WI. C1–6 were T1WI.

The process of patient screening and inclusion, as well as the collection of information, has been previously described^10^. The information was electronically stored in a secure database (Real Data EDC system) for subsequent analysis. Patients were classified as mild and severe CDA, and the latter referred to those who meet any of the following criteria: (1) experiencing symptomatic hemorrhage by the age of 18; (2) experiencing more than one symptomatic hemorrhage event; (3) showing more than 25 lesions on SWI, or more than 5 lesions on T2-weighted images (Fig. 1). Adjudication of the classification was performed by the senior author of this study, who was blinded from any information on the biomarker levels.

### Assessment of plasma biomarker levels

Candidate biomarkers were selected based on their reported roles in angiogenesis, inflammation, endothelial cell integrity and permeability, cell adhesion, and extracellular matrix remodeling (Supplemental Table 1)^8,15–18^. All blood samples were collected using standard clinical 5ml heparinized vacutainer tubes. The use of heparinized plasma to quantify biological compounds was in agreement with clinical practice and the instructions provided by the bio-assay kit manufacturer (Milliplex Human Cytokine kit; Millipore Corp., Billerica, MA). Fasting was not required because the clinical visits were conducted at various times of the day. For plasma isolation, 5 ml of heparinized blood was centrifuged at 3000 rpm for 5 min. The supernatant plasma was aliquoted into microcentrifuge tubes (500 μl) and stored at − 80°C. Sixty-seven plasma biomarkers were assessed using a commercial multiplex bead immunoassay kit (Milliplex Human Cytokine Kit; Millipore Corp., Billerica, MA, USA). Measurements were performed using a Bio-Plex suspension array system (Bio-Plex200; Bio-Rad, Hercules, CA, USA). All the plasma samples used in this study were processed and tested in the same batch. The concentration levels of plasma factors below the limit of detection were replaced by the limit of detection divided by the square root of 2.

### Statistical analysis

First, normal analysis was performed using the Shapiro-Wilk test on quantitative data, including demographic data, clinical characteristics, and plasma factors. Most of the data showed a skewed distribution; medians and interquartile ranges were used to describe these data. Quantitative data were compared between two groups using the Mann-Whitney U test. Categorical variables were descriptively summarized as numbers (%), and distribution differences between groups were calculated using the chi-square test or Fisher’s test. The correlations between plasma factors were analysed with Spearman’s rank correlation coefficient. Second, logistic regression analyses were performed to determine the associations between plasma factors and the risk of CCM occurrence and severe CDA. Variables with *P* < 0.05 in the univariate analysis were selected for inclusion in a multivariate logistic model (Backwald: Wald). Third, receiver operating characteristic (ROC) curves were generated, and the area under the curve (AUC) was calculated for each plasma factor and in linear combination (discriminant score). The best model was defined as the one for which the AUC value was greatest, and the optimal cut-off value which provides the best tradeoff between sensitivity and specificity was found using the Youden index method. Finally, generalized linear mixed models (GLMM) were used for sensitivity analysis, defining the individual level as low level 1 and pedigree level as high level 2. Analyses were performed using SPSS (version 26.0; R version 4.1.0) and GraphPad Prism (version 8.0). Two-tailed P-values < 0.05 were considered statistically significant.

## Results

**Figure 2:**
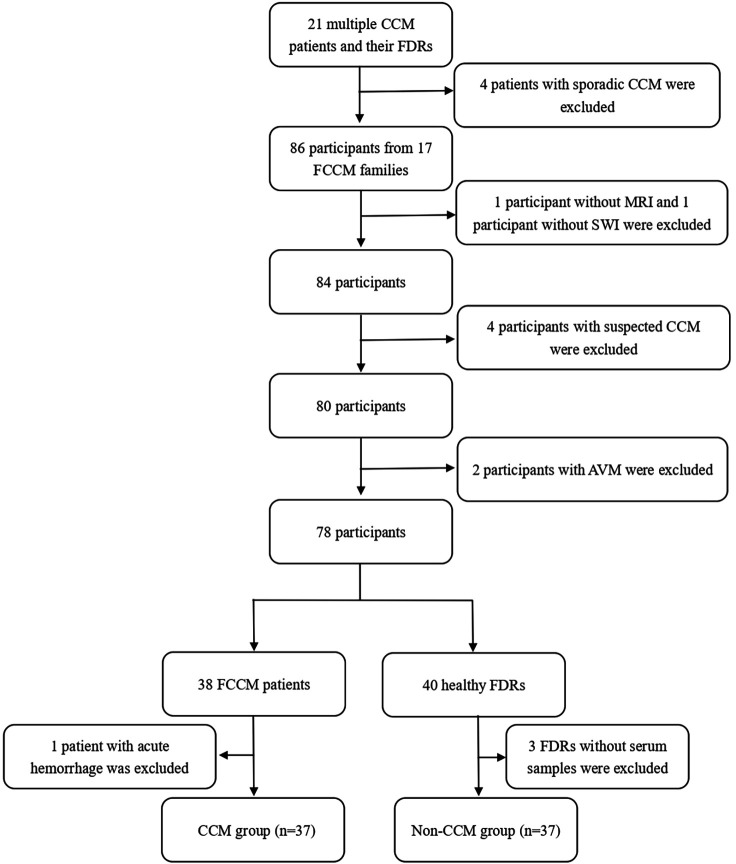
Patients and First-level relatives (FLRs) Screening Flowchart

Twenty-one multiple CCM patients and their FDRs were screened to identify FCCM families. Based on the MRI scanning, gene testing, and family history survey, 4 patients with neither germline mutations in the *CCM1/KRIT1, CCM2/malcavernin*, or *CCM3/PDCD10* genes nor a family history of CCM were defined as sporadic CCM cases. The remaining 17 multiple CCM patients were identified as index cases of FCCM families. The initial symptoms noted in the 17 probands were hemorrhage in 5 patients (29.4%), epilepsy in 5 patients (29.4%), headache in 2 patients (11.8%), functional neurological deficit (FND) in 1 patient (5.9%), and fever in 1 patient (5.9%), and incidentally spotted by MRI in 3 patients (17.6%). Among a total of 86 participants from the 17 FCCM families, two without intact MRI images, four with suspected CCMs and two with arteriovenous malformations on SWI, one with acute intracerebral hemorrhage on MRI and three who refused to draw blood were all excluded. Finally, the plasma biomarker candidates were examined in the CCM group consisted of 37 FCCM patients and the non-CCM group consisted of 37 healthy FDRs (Fig. 2).

**Figure 3:**
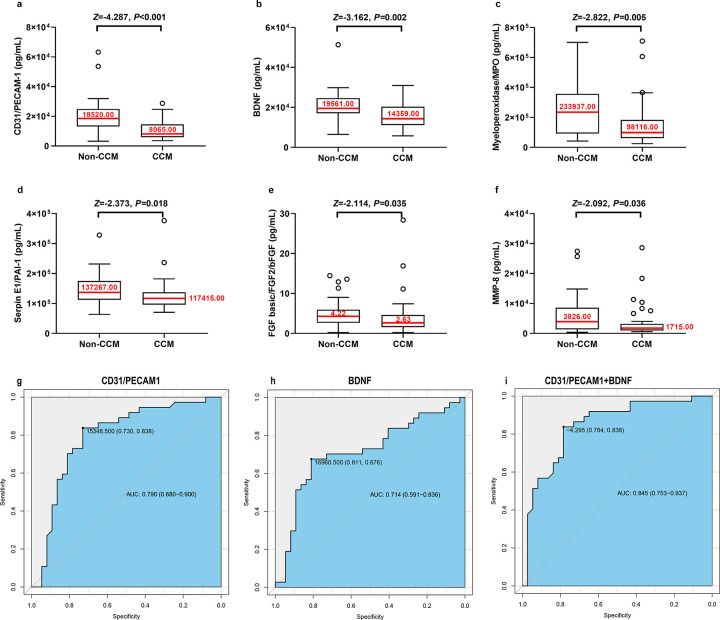
a-f. Lower CD31 (*P*<0.001), BDNF (*P*=0.002), Myeloperoxidase/MPO (*P*=0.005), Serpin E1/PAI-1 (*P*=0.018), FGF basic/FGF2/bFGF (*P*=0.035) and MMP-8 (*P*=0.036) were associated with the occurrence of CCM in FCCM family determined between 37 FCCM patients and 37 healthy FLRs by Mann-Whitney U test. g-i. The receiver operating characteristic (ROC) curve generated using plasma factors distinguished the CCM patients from non-CCMs FLRs. (g) The ROC curve of BDNF. (h) The ROC curve of CD31/PECAM-1. (i) The ROC curve generated using the combination of plasma levels of BDNF and CD31/PECAM-1 to differentiate CCM patients from non-CCMs FLRs.

Baseline epidemiological and clinical characteristics of study participants were showed in Supplemental Table 2. The differences in baseline characteristics, between patients with FCCM and healthy FDRs were not significant. The details of the comparison of plasma factor levels between patients with FCCM and healthy FDRs are listed in Supplemental Table 1. Low CD31 (*P* < 0.001), BDNF (*P* = 0.002), myeloperoxidase/MPO (*P* = 0.005), serpin E1/PAI-1 (*P* = 0.018), FGF basic/FGF2/bFGF (*P* = 0.035), and MMP-8 (*P* = 0.036) levels were associated with the occurrence of FCCM, as assessed from the Mann-Whitney U test (Fig. 3a–f). However, according to the multivariate logistic regression analysis, only low CD31 [OR (95%CI) 0.193(0.0780–0.478); *P* < 0.001] and BDNF levels [OR (95%CI) 0.441(0.231–0.844); *P* = 0.013] were independent risk factors for the occurrence of FCCM (Table 1). The accuracies to distinguish patients with FCCM from healthy FDRs were considered as “fair” when using CD31 (AUC = 0.790, sensitivity 0.838, specificity 0.730, cutoff point 15348.50 pg/mL) and BDNF as biomarkers (AUC = 0.714, sensitivity 0.676, specificity 0.811, cutoff point 16960.50 pg/mL) (Fig. 3g–i). Further analysis showed that the best model (Eq. 1) was achieved by combining the results for CD31 and BDNF (AUC = 0.845, sensitivity 0.838, specificity 0.784, cutoff score − 4.295). Eq. 1: Discriminant score= [CD31PECAM1]/(−6436.353) +[BDNF] / (−8890.952); the discriminant score was calculated based on multivariate regression coefficients. The GLMM results showed that the family aggregation effect (intraclass correlation coefficient [ICC] = 0.042, *P* = 0.660) was not significant for plasma factors. However, low CD31 [OR (95%CI) 0.187 (0.068–0.514)] and BDNF [OR (95%CI) 0.431(0.212–0.875)] levels were still associated with the occurrence of FCCM when analyzed using the GLMM. The discriminant ability of the discriminant score established using the GLMM (AUC = 0.846, sensitivity = 0.838, specificity = 0.784) agreed with and supported the logistic regression results mentioned above.

**Figure 4:**
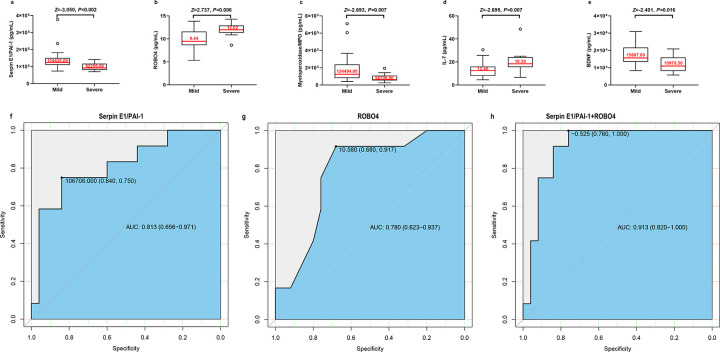
a-e. Lower Serpin E1/PAI-1 (*P*=0.002), Myeloperoxidase/MPO (*P*=0.007) and BDNF (*P*=0.016), but higher IL-7 (*P*=0.007) and ROBO4 (*P*=0.006) were associated with the severe CDA in FCCM patients determined between 12 FCCM patients with severe CDA and 25 FCCM patients with mild CDA by Mann-Whitney U test. f-h. The receiver operating characteristic (ROC) curve generated using plasma factors to distinguish patients with the severe or mild chronic disease aggressiveness in CCM patients. (f) The ROC curve of Serpin E1/PAI-1. (g) The ROC curve of ROBO4. (h) The ROC curve generated using the combination of plasma levels of Serpin E1/PAI-1 and ROBO4.

According to the criteria of CDA, 12 patients were dichotomized into the severe CDA group and the other 25 into the mild CDA group. The differences in baseline characteristics, including sex (*P* = 0.893), body mass index (*P* = 0.897), lifestyle habits (smoking *P* = 0.328, drinking *P* = 0.582), underlying diseases (hypertension, *P* = 0.819; diabetes, *P* = 0.324; and hyperlipidemia, *P* = 1.000), and mutation in *CCM* genes (*P* = 0.225), between the two groups were not significant. However, the mean age was higher in the severe CDA group than in the mild group (47.50 [38.00, 58.50] vs.34.00 [9.00, 43.00] years, *P* = 0.008) (Supplemental Table 3). The details of the plasma biomarker comparisons between the severe and mild CDA groups are listed in Supplemental Table 4. Low serpin E1/PAI-1 (*P* = 0.002), myeloperoxidase/MPO (*P* = 0.007), and BDNF (*P* = 0.016) but high IL-7 (*P* = 0.007) and ROBO4 (*P* = 0.006) levels were associated with severe CDA in FCCM patients, as determined by the Mann-Whitney U test (Fig. 4a–e). However, according to the multivariate logistic regression analysis, only low serpin E1/PAI-1 [OR (95%CI) 0.026(0.002–0.437); *P* = 0.011] and highROBO4 [OR (95%CI) 7.624(1.548–37.558); *P* = 0.013] were independent risk factors for severe CDA in patients with FCCM (Table 2). The accuracies to distinguish severe CDA FCCM patients were considered as “fair” when using ROBO4 (AUC = 0.780, sensitivity 0.917, specificity 0.680, cutoff point 10.58 pg/mL) and as “good” when using E1/PAI-1 as markers (AUC = 0.813, sensitivity 0.750, specificity 0.840, cutoff point 106706.00 pg/mL) (Fig. 4f–h). Further analysis showed that the best model (Eq. 2) was achieved by combining the results of E1/PAI-1 and ROBO4 (AUC = 0.913, sensitivity = 1.000, specificity = 0.760, cutoff score − 0.525). Eq. 2: Discriminant score= [SerpinE1PAI1]/(−14508.416)+[ROBO4]/(1.423); the discriminant score was calculated based on multivariate regression coefficients. The GLMM results showed that the family aggregation effect (intraclass correlation coefficient ICC = 0.412, *P* = 0.592) was not significant for plasma factors. However, low E1/PAI-1 [OR (95%CI) 0.012 (0.000–0.491)] and high ROBO4 levels [OR (95%CI) 11.797 (1.483–93.844)] were still associated with severe CDA in patients with FCCM when analyzed using the GLMM. The discriminant ability of the discriminant score established using the GLMM (AUC = 0.915, sensitivity = 1.000, specificity = 0.822) agreed with and supported the logistic regression results mentioned above.

**Figure 5:**
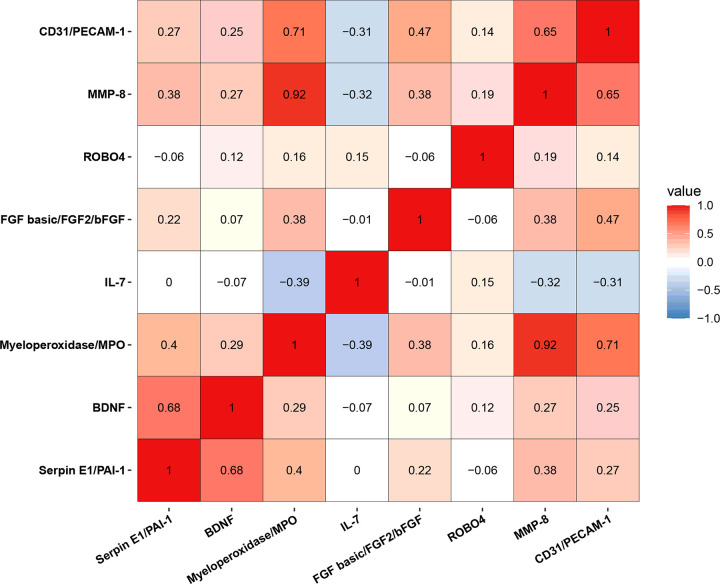
The correlation analysis for candidate factors revealed Strong correlation between most factors, especially between MPO and MMP8 (r = 0.92, 95% CI: 0.87–0.95) and MPO and CD31 (r = 0.71, 95% CI: 0.57–0.81).

The correlation analysis for 67 serum factors were independent of each other or showed a weak correlation (Supplementary Excel). However, among the candidate factors, strong correlations were found between MPO and MMP8 (r = 0.92, 95% CI: 0.87–0.95) and MPO and CD31 (r = 0.71, 95% CI: 0.57–0.81; Fig. 5).

## Discussion

The plasma levels of molecules reflecting proliferative dysangiogenesis, blood-brain barrier hyperpermeability, inflammatory/immune processes, and measures of vascular permeability and iron deposition on magnetic resonance imaging are important biomarkers correlated with CCM occurrence and bleeding^6–8,19^. In 2021, the American NIH/NINDS published the protocol of their study on biomarkers of cerebral cavernous angioma with symptomatic hemorrhage (CASH) to optimize these biomarkers to accurately diagnose CCM with symptomatic hemorrhage (R01NS114552)^20^. However, until now, none of the biomarkers have shown an association with de novo lesion genesis and symptomatic hemorrhage in FCCM cases. Patients with FCCM with small non-aggressive multiple lesions are usually managed conservatively^2^. Nevertheless, invasive intervention in multifocal FCCM cases, although controversial, can be justifiable when these lesions become symptomatic or develop. Thus, development and validation of a biomarker model to predict the occurrence and clinical activity of FCCMs may play a direct role in selecting patients with FCCM for aggressive therapies and in the stratification of cohorts in future clinical trials. To the best of our knowledge, this study is the first to investigate the differences in plasma biomarker levels between FCCM patients and their healthy FDRs and between FCCM patients with and without severe CDA in a large panel of 67 candidate plasma biomarkers. We found that low CD31and BDNF levels were independent risk factors associated with the occurrence of FCCM; the best model was achieved by combining the results of CD31 and BDNF (AUC = 0.845). Furthermore, serpin E1/PAI-1 and high ROBO4 levels were independent risk factors associated with severe CDA in patients with FCCM, and the best model was achieved by combining the results of E1/PAI-1 and ROBO4 (AUC = 0.913).

Patients and healthy FDRs have similar genetic backgrounds and lifestyles. Therefore, the significant differences in plasma concentrations of CD31 and BNDF might be related to the pathogenesis of CCM. The past two decades have witnessed a remarkable enhancement of our understanding of the pathogenesis of this vascular disease. In FCCM, lesion formation is initiated by a somatic mutation in the *CCM* gene, resulting in biallelic loss of function, and a secondary somatic gain-of-function mutation in PIK3CA (phosphatidylinositol-4,5-bisphosphate 3-kinase catalytic subunit alpha), which fuels lesion growth^21,22^. As a ligand, BDNF-induced TrkB activation of the PI3-kinase and Akt-mTOR pathway may mediate a reduction in EC–cell contacts and in normal EC apoptosis, leading to the development of FCCMs^23,24^. BDNF is best characterized for its pro-survival and differentiative effects on neurons; additionally, studies have uncovered the contribution of the cerebral microvasculature to BDNF production in the brain^25,26^. BDNF deficiency can lead to a reduction in endothelial cell-cell contacts^27^. However, whether the reduction in plasma BDNF concentration in FCCM patients is due to excessive utilization of BDNF remains unknown and could not be clarified in this study. CD31 is a cell-cell adhesion glycoprotein that belongs to the immunoglobulin superfamily^28,29^. CD31 is not only an endothelial marker but is also involved in the regulation of endothelial cell-cell interactions and angiogenesis^30,31^. The observation that the anti-CD31 antibody blocks the initiation of EC-EC contact suggests an important role of CD31 in maintaining the structural integrity of the EC layer^32^. A previous study showed that murine hemangioendothelioma cells expressed lower levels of CD31 and other components of adherent junctions than wild-type brain endothelial cells (BECs)^33^. BECs with CD31 deficiency (isolated from CD31-knockout mice) would in part mimic the phenotypes of cultured hemangioendothelioma cells, exhibiting an “overriding” morphology, a higher secondary proliferation rate due to the loss of contact inhibition, and a reduced level of apoptosis^33,34^.It was demonstrated that in addition to disrupted tight junction integrity, CD31-deficiency resulted in a perturbation of adhesion molecule-mediated signaling, which affected proliferation and suppressed apoptosis^33,34^, suggesting one of the molecular mechanisms underlying endothelial cell behaviors when vascular anomalies develop in hemangiomas. Owing to the popularity of MRI, the use of CD31 and BDNF scores to distinguish patients with FCCM from healthy FDRs has little clinical significance. However, this score may serve as a biomarker for patient screening and a warning for FCCM occurrence at an early stage. Whether CD31 and BDNF are related to the mechanism of FCCM and whether they can serve as targets for medical intervention requires further research.

An early age of lesion onset, multiple hemorrhages, and increased lesion burden have been defined as severe chronic disease severity in various studies^6,35^. Acute hemorrhage and epileptic seizures have a significant influence on plasma factor concentrations; therefore, they were excluded from this study. The molecular mechanisms influencing chronic disease severity have not yet been elucidated, and there are currently no known peripheral blood biomarkers that reflect or predict disease aggressiveness in the FCCM population. Recently, several plasma factors, including 25-hydroxyvitamin D, non-HDL cholesterol, IL-2, INF-γ, TNF-α, IL-1β, MMP-2, and − 9, intercellular adhesion molecule-1, VEG, and endoglin, have been proposed to monitor disease severity and the course of sporadic CCMs^7,36^.Furthermore, the weighted linear combination of soluble CD14, IL-1β, VEGF, and soluble ROBO4 can be used to predict symptomatic ICH or lesion expansion^8^. ROBO4 is an endogenous inhibitor of VEGF signaling expressed by vascular endothelial cells^37,38^.This protein has been shown to dynamically maintain vascular network stability during pathological angiogenesis and proinflammatory processes^39,40^.In the FCCM cohort, an increase in plasma ROBO4 levels may reflect pro-inflammatory processes enhancing endothelial permeability, consistent with its prognostic association with CCM bleeding and growth^8^.E1/PAI-1 is a fast-acting inhibitor of tissue and urokinase plasminogen activators (tPA and uPA)^41^. PAI-1 controls the clot lysis triggered by tPA-activated plasminogen. PAI-1 deficiency is characterized by hyperfibrinolysis, which results in frequent bleeding episodes. Patients with this condition form normal blood clots, which are quickly lysed by unopposed tPA-activated plasmin. Spontaneous bleeding is rare in PAI-1 deficient patients, but moderate hemorrhage of the knees, elbows, nose, and gums can be triggered by mild trauma^42^. In the context of FCCM, lower plasma levels of E1/PAI-1 maybe associated with more frequent cerebral bleeding and a larger hemorrhage volume after ictus. However, the role of E1/PAI-1 in CCM lesion development and hemorrhage requires further investigation. In our study, age was higher in the severe CDA group than in the mild CDA group (*P* = 0.008) in the univariate analysis (Supplemental Table 3). Variables with *P* < 0.05 in the univariate analysis, including age, serpin E1/PAI-1, ROBO4, IL-7, and BDNF, were selected for inclusion in a multivariate logistic model. However, age was eliminated from the backward model, and only Serpin E1/PAI-1 and ROBO4 were retained in the final model. In addition, we constructed a new model (age + serpin E1/PAI-1 + ROBO4) to re-evaluate the effect of age on disease severity. A model algorithm was established based on the results of the multivariate logistic model. Equation: Discriminant score = age × 0.06012+ (Serpin E1/PAI-1)* (−0.00005) +(ROBO4) × 0.78396. The AUC of the new model was 0.940, which was higher than that of the model without age (serpin E1/PAI-1 + ROBO4 [AUC:0.913]); however, the difference was not statistically significant (Z = − 0.831, *P* = 0.406). It can be observed that the improvement in age for the model was limited. Overall, we found that the combination of E1/PAI-1 and ROBO4 was the best model to distinguish FCCM patients with mild and severe CDA; therefore, it may be helpful in the prognostication and stratification of FCCM cases in future clinical trials.

### Limitations

Our single-site study did not exclude a referral bias. Future multi-site studies are needed to better control and circumvent these potential biases in a sufficient number of cases, if not all. In addition, the correlations herein do not imply a specific causality related to CCM. However, they resulted in a cogent hypotheses about the mechanism of disease occurrence and progression that can be pursued in future laboratory and clinical studies. Finally, the limitations of the assay methodologies and batch effects must be considered in clinical practice. However, in this study, we investigated the differences in plasma biomarker levels between FCCM patients and their FDRs and between FCCM patients with and without CDA in a large panel of 67 plasma biomarkers. These results may play a direct role in selecting FCCM patients for aggressive therapies and may be critical for defining biological targets for medical therapies.

## Conclusion

In the FCCM family, plasma concentrations of CD31 and BDNF seems to be lower in FCCM patients; low serpin E1/PAI-1 and high ROBO4 concentrations may be correlated with high lesion burden and risk of recurrent bleeding. Future multi-site studies with a larger number of cases are needed to confirm our findings.

## Figures and Tables

**Table 1:** Logistic regression analysis of the association between differential expression levels of plasma factors and CCM occurrence.

Variables	Univariate		Multivariate	
	OR (95%CI)	P	OR (95%CI)	P
CD31/PECAM1 (per SD)	0.195(0.083–0.456)	< 0.001	0.193(0.0780–0.478)	< 0.001
BDNF (per SD)	0.433(0.235–0.796)	0.007	0.441(0.231–0.844)	0.013
Myeloperoxidase/MPO (per SD)	0.520(0.300–0.900)	0.019	-	-
Serpin E1/PAI-1 (per SD)	0.622(0.358–1.079)	0.091	-	-
FGF basic/FGF2/bFGF (per SD)	0.828(0.510–1.345)	0.446	-	-
MMP-8 (per SD)	0.654(0.381–1.122)	0.123	-	-

Abbreviations: OR, odds ratio; CI, confidence interval; SD, standard deviation. *: backward method was used to screen variables in multivariate logistic regression analysis. The variables entered initially in the model include CD31/PECAM-1, BDNF, and Myeloperoxidase/MPO.

## Data Availability

Data is provided within the manuscript or supplementary information files.

## References

[1] Carrión-Penagos J, Zeineddine H, Polster S, Subclinical imaging changes in cerebral cavernous angiomas during prospective surveillance. Journal of neurosurgery. 2020;134(3):1147–1154. doi:10.3171/2020.1.Jns193479 32244216 PMC7541547

[2] Zafar A, Quadri S, Farooqui M, Familial Cerebral Cavernous Malformations. Stroke. 2019;50(5):1294–1301. doi:10.1161/strokeaha.118.022314 30909834 PMC6924279

[3] Morrison L, Akers A. Cerebral Cavernous Malformation, Familial. GeneReviews^™^; 1993.

[4] Li X, Fisher O, Boggon T. The cerebral cavernous malformations proteins. Oncotarget. 2015;6(32):32279–80. doi:10.18632/oncotarget.5443 26356566 PMC4741677

[5] Al-Shahi Salman R, Hall J, Horne M, Untreated clinical course of cerebral cavernous malformations: a prospective, population-based cohort study. The Lancet Neurology. 2012;11(3):217 – 24. doi:10.1016/s1474-4422(12)70004-2 22297119 PMC3282211

[6] Choquet H, Pawlikowska L, Nelson J, Polymorphisms in inflammatory and immune response genes associated with cerebral cavernous malformation type 1 severity. Cerebrovascular diseases (Basel, Switzerland). 2014;38(6):433 – 40. doi:10.1159/000369200 25472749 PMC4297571

[7] Girard R, Zeineddine H, Fam M, Plasma Biomarkers of Inflammation Reflect Seizures and Hemorrhagic Activity of Cerebral Cavernous Malformations. Translational stroke research. 2018;9(1):34–43. doi:10.1007/s12975-017-0561-3 28819935 PMC5750099

[8] Girard R, Zeineddine H, Koskimäki J, Plasma Biomarkers of Inflammation and Angiogenesis Predict Cerebral Cavernous Malformation Symptomatic Hemorrhage or Lesional Growth. Circulation research. 2018;122(12):1716–1721. doi:10.1161/circresaha.118.312680 29720384 PMC5993629

[9] Lazzaroni F, Meessen J, Sun Y, Circulating biomarkers in familial cerebral cavernous malformation. EBioMedicine. Jan 2024;99:104914. doi:10.1016/j.ebiom.2023.104914 38113759 PMC10767159

[10] Li C, Zhuo L, Kang Y, Prevalence, genetic and clinical characteristics in first-degree relatives of patients with familial cerebral cavernous malformations in China. Stroke Vasc Neurol. May 15 2024;doi:10.1136/svn-2023-003004 PMC11877442 38749536

[11] de Champfleur N, Langlois C, Ankenbrandt W, Magnetic resonance imaging evaluation of cerebral cavernous malformations with susceptibility-weighted imaging. Neurosurgery. 2011;68(3):641–7; discussion 647–8. doi:10.1227/NEU.0b013e31820773cf 21164377

[12] Campbell P, Jabbour P, Yadla S, Awad I. Emerging clinical imaging techniques for cerebral cavernous malformations: a systematic review. Neurosurgical focus. 2010;29(3):E6. doi:10.3171/2010.5.Focus10120 PMC3708641 20809764

[13] de Souza J, Domingues R, Cruz L, Domingues F, Iasbeck T, Gasparetto E. Susceptibility-weighted imaging for the evaluation of patients with familial cerebral cavernous malformations: a comparison with t2-weighted fast spin-echo and gradient-echo sequences. AJNR American journal of neuroradiology. 2008;29(1):154–8. doi:10.3174/ajnr.A0748 17947370 PMC8119083

[14] Cuschieri SJSjoa. The STROBE guidelines. 2019;13:S31–S34. doi:10.4103/sja.SJA_543_18 PMC6398292 30930717

[15] Claesson-Welsh L, Dejana E, McDonald D. Permeability of the Endothelial Barrier: Identifying and Reconciling Controversies. Trends in molecular medicine. 2021;27(4):314–331. doi:10.1016/j.molmed.2020.11.006 33309601 PMC8005435

[16] Chai P, Yu J, Li Y, Shi Y, Fan X, Jia R. High-throughput transcriptional profiling combined with angiogenesis antibody array analysis in an orbital venous malformation cohort. Experimental eye research. 2020;191:107916. doi:10.1016/j.exer.2020.107916 31926133

[17] Retta S, Glading A. Oxidative stress and inflammation in cerebral cavernous malformation disease pathogenesis: Two sides of the same coin. The international journal of biochemistry & cell biology. 2016;81:254–270. doi:10.1016/j.biocel.2016.09.011 27639680 PMC5155701

[18] Zhao M, Peng C, Li L, Chen L, Zhang H. Circulating Treg cells from patients with cerebral aneurysms displayed deficiency in ICOS expression and function. Clinical and experimental pharmacology & physiology. 2020;47(12):1923–1931. doi:10.1111/1440-1681.13388 32726458

[19] Chohan M, Marchiò S, Morrison L, Emerging Pharmacologic Targets in Cerebral Cavernous Malformation and Potential Strategies to Alter the Natural History of a Difficult Disease: A Review. JAMA neurology. 2019;76(4):492–500. doi:10.1001/jamaneurol.2018.3634 30476961

[20] Girard R, Li Y, Stadnik A, A Roadmap for Developing Plasma Diagnostic and Prognostic Biomarkers of Cerebral Cavernous Angioma With Symptomatic Hemorrhage (CASH). Neurosurgery. 2021;88(3):686–697. doi:10.1093/neuros/nyaa478 33469662 PMC7884145

[21] Ren A, Snellings D, Su Y, PIK3CA and CCM mutations fuel cavernomas through a cancer-like mechanism. Nature. 2021;594(7862):271–276. doi:10.1038/s41586-021-03562-8 33910229 PMC8626098

[22] Snellings D, Hong C, Ren A, Cerebral Cavernous Malformation: From Mechanism to Therapy. Circulation research. 2021;129(1):195–215. doi:10.1161/circresaha.121.318174 34166073 PMC8922476

[23] Sha’ari H, Haerian B, Baum L, Association of BDNF Polymorphisms with the Risk of Epilepsy: a Multicenter Study. Molecular neurobiology. 2016;53(5):2869–2877. doi:10.1007/s12035-015-9150-1 25876511

[24] Kermani P, Hempstead B. Brain-derived neurotrophic factor: a newly described mediator of angiogenesis. Trends in cardiovascular medicine. 2007;17(4):140–3. doi:10.1016/j.tcm.2007.03.002 17482097 PMC2268985

[25] Guo S, Kim W, Lok J, Neuroprotection via matrix-trophic coupling between cerebral endothelial cells and neurons. Proceedings of the National Academy of Sciences of the United States of America. 2008;105(21):7582–7. doi:10.1073/pnas.0801105105 18495934 PMC2396701

[26] Monnier A, Prigent-Tessier A, Quirié A, Brain-derived neurotrophic factor of the cerebral microvasculature: a forgotten and nitric oxide-dependent contributor of brain-derived neurotrophic factor in the brain. Acta physiologica (Oxford, England). 2017;219(4):790–802. doi:10.1111/apha.12743 27364224

[27] Donovan M, Lin M, Wiegn P, Brain derived neurotrophic factor is an endothelial cell survival factor required for intramyocardial vessel stabilization. Development (Cambridge, England). 2000;127(21):4531–40. doi:10.1242/dev.127.21.4531 11023857

[28] DeLisser H, Newman P, Albelda S. Molecular and functional aspects of PECAM-1/CD31. Immunology today. 1994;15(10):490–5. doi:10.1016/0167-5699(94)90195-3 7945775

[29] Watt S, Gschmeissner S, Bates P. PECAM-1: its expression and function as a cell adhesion molecule on hemopoietic and endothelial cells. Leukemia & lymphoma. 1995;17:229 – 44. doi:10.3109/10428199509056827 8580791

[30] Albelda S, Oliver P, Romer L, Buck C. EndoCAM: a novel endothelial cell-cell adhesion molecule. The Journal of cell biology. 1990;110(4):1227–37. doi:10.1083/jcb.110.4.1227 2182647 PMC2116064

[31] Matsumura T, Wolff K, Petzelbauer P. Endothelial cell tube formation depends on cadherin 5 and CD31 interactions with filamentous actin. Journal of immunology (Baltimore, Md : 1950). 1997;158(7):3408–16. 9120301

[32] Albelda S, Muller W, Buck C, Newman P. Molecular and cellular properties of PECAM-1 (endoCAM/CD31): a novel vascular cell-cell adhesion molecule. The Journal of cell biology. 1991;114(5):1059–68. doi:10.1083/jcb.114.5.1059 1874786 PMC2289123

[33] Tsuneki M, Madri J. Adhesion molecule-mediated hippo pathway modulates hemangioendothelioma cell behavior. Molecular and cellular biology. 2014;34(24):4485–99. doi:10.1128/mcb.00671-14 25266662 PMC4248725

[34] Tsuneki M, Madri J. CD44 regulation of endothelial cell proliferation and apoptosis via modulation of CD31 and VE-cadherin expression. The Journal of biological chemistry. 2014;289(9):5357–70. doi:10.1074/jbc.M113.529313 24425872 PMC3937614

[35] Al-Holou W, O’Lynnger T, Pandey A, Natural history and imaging prevalence of cavernous malformations in children and young adults. Journal of neurosurgery Pediatrics. 2012;9(2):198–205. doi:10.3171/2011.11.Peds11390 22295927

[36] Girard R, Khanna O, Shenkar R, Peripheral plasma vitamin D and non-HDL cholesterol reflect the severity of cerebral cavernous malformation disease. Biomarkers in medicine. 2016;10(3):255 – 64. doi:10.2217/bmm.15.118 26861901 PMC4926533

[37] Chen X, Chen X, Xu Y, Association of six CpG-SNPs in the inflammation-related genes with coronary heart disease. Human genomics. 2016:21. doi:10.1186/s40246-016-0067-1 27461004 PMC4965732

[38] Becerra A, Probst C, Tejani M, Evaluating the Prognostic Role of Elevated Preoperative Carcinoembryonic Antigen Levels in Colon Cancer Patients: Results from the National Cancer Database. Annals of surgical oncology. 2016;23(5):1554–61. doi:10.1245/s10434-015-5014-1 26759308

[39] Gao Y, Zhao Z, Yang L, Arsenic exposure assists ccm3 genetic polymorphism in elevating blood pressure. Oncotarget. 2018;9(4):4915–4923. doi:10.18632/oncotarget.23518 29435151 PMC5797022

[40] Shenkar R, Shi C, Rebeiz T, Exceptional aggressiveness of cerebral cavernous malformation disease associated with PDCD10 mutations. Genetics in medicine : official journal of the American College of Medical Genetics. 2015;17(3):188–196. doi:10.1038/gim.2014.97 25122144 PMC4329119

[41] Vaughan D. PAI-1 and atherothrombosis. Journal of thrombosis and haemostasis : JTH. 2005;3(8):1879–83. doi:10.1111/j.1538-7836.2005.01420.x 16102055

[42] Jankun J, Skrzypczak-Jankun E. Plasminogen activator inhibitor with very long half-life (VLHL PAI-1) can reduce bleeding in PAI-1-deficient patients. Cardiovascular & hematological disorders drug targets. 2013;13(2):144 – 50. doi:10.2174/1871529x11313020007 23988002

